# Endoscopic injection sclerotherapy with ligation for esophageal varices using single-use endoscope: Report of a video case

**DOI:** 10.1055/a-2598-4466

**Published:** 2025-05-26

**Authors:** Kazunori Nagashima, Yasunori Inaba, Ken Kashima, Yasuhito Kunogi, Fumi Sakuma, Akira Yamamiya, Atsushi Irisawa

**Affiliations:** 1 Department of Gastroenterology, Dokkyo Medical University School of Medicine, Mibu, Japan

In recent years, single-use endoscopes ( Fig. 1 ) have been used for diagnosis and treatment 1 2 . They have a wide range of motion. Moreover, the scope tip is translucent during fluoroscopy ( Fig. 2 ). Their wide range of motion makes endoscopic variceal ligation (EVL) easy to perform. Additionally, because of the highly radiolucent structure of the scope tip, the flow of the injected sclerosing agent supplemented with contrast medium can be observed clearly during endoscopic injection sclerotherapy (EIS). This case report includes the first video recording of the use of a single-use endoscope for endoscopic injection sclerotherapy with ligation (EISL) for esophageal varices.

**Fig. 1:**
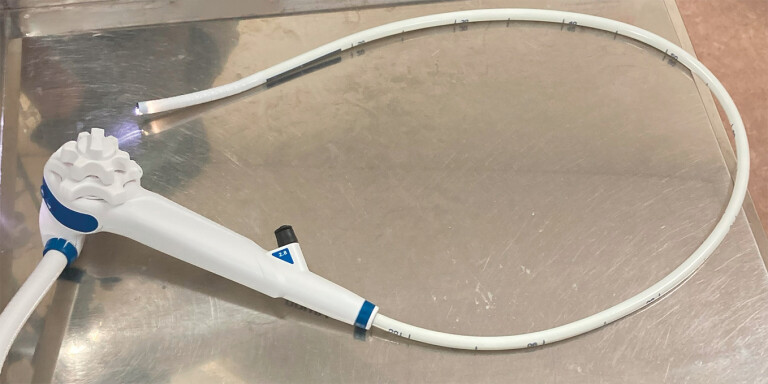
Single-use endoscope (aScope Gastro; Ambu A/S, Tokyo, Japan).

**Fig. 2:**
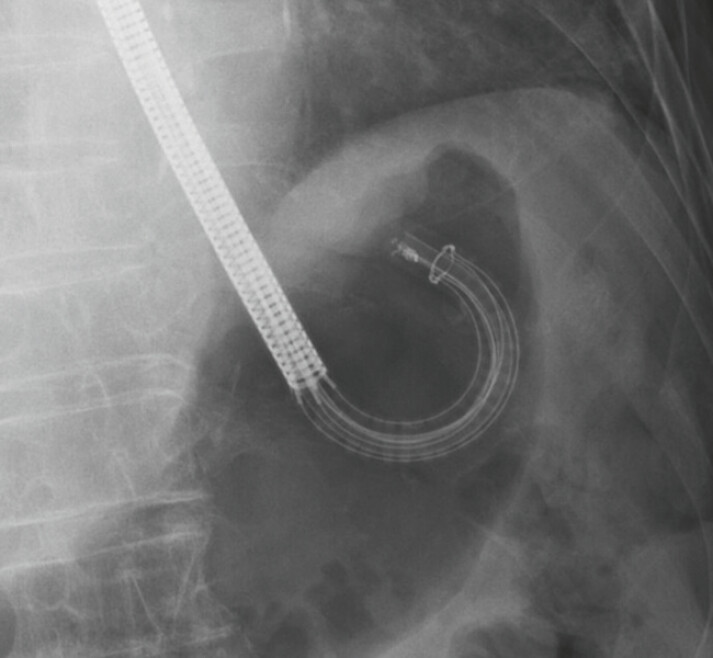
Single-use endoscopes have a wide range of motion. The scope tip is translucent during fluoroscopy.

This video presents a typical case ( Video 1 ). The 77-year-old man had alcoholic cirrhosis and esophageal varices ( Fig. 3 ). A single-use endoscope (aScope Gastro; Ambu A/S, Tokyo, Japan) equipped with a balloon and an EVL device at its tip was used ( Fig. 4 ). After inflating a balloon attached to the endoscope tip, the varices were punctured using a 25-gauge needle (Varixer; TOP Corp., Tokyo, Japan). Use of the balloon prevents migration of sclerosant into the drainage flow. Sclerosant (ethanolamine oleate) was injected into the feeder while we confirmed the injection using fluoroscopy. The tip structure facilitates checking whether sclerosant is flowing into the drainage flow ( Fig. 5 ). This confirmation can prevent an embolism into the general circulation. After EIS, EVL was performed for the puncture hole. No adverse event occurred.

This report is the first video case report describing performance of endoscopic injection sclerotherapy with ligation for endoscopy using a single-use endoscope. This scope is useful for varix treatment using fluoroscopy.Video 1

**Fig. 3:**
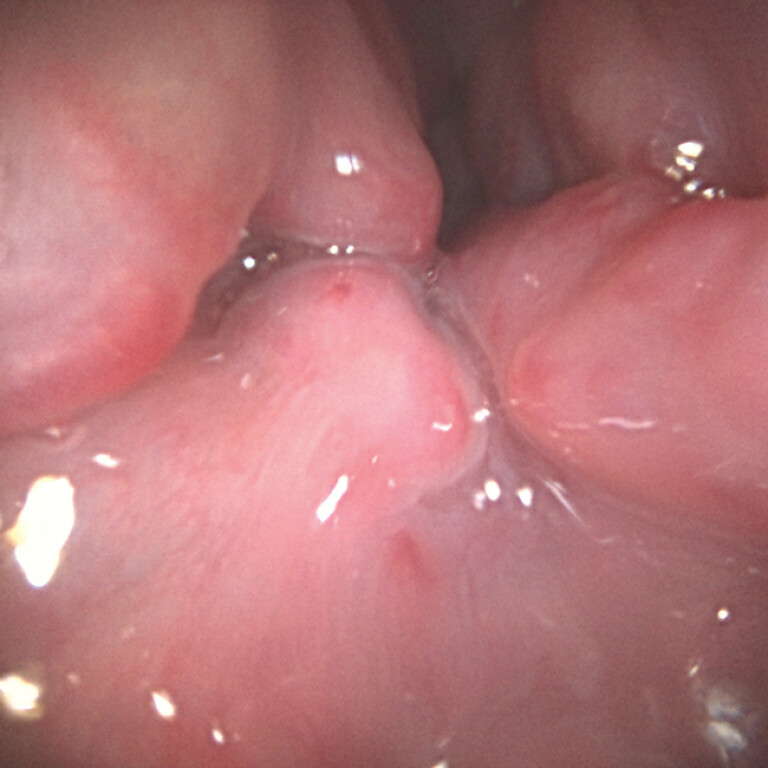
Varices showed strong development.

**Fig. 4:**
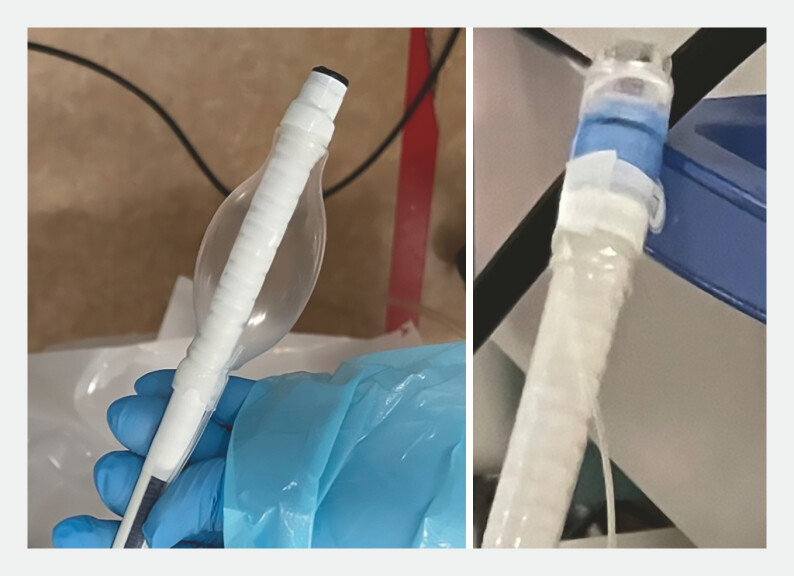
A balloon and endoscopic variceal ligation (EVL) device attached to the endoscope tip can be inflated to prevent embolization of the drainage flow. EVL can be performed quickly.

**Fig. 5:**
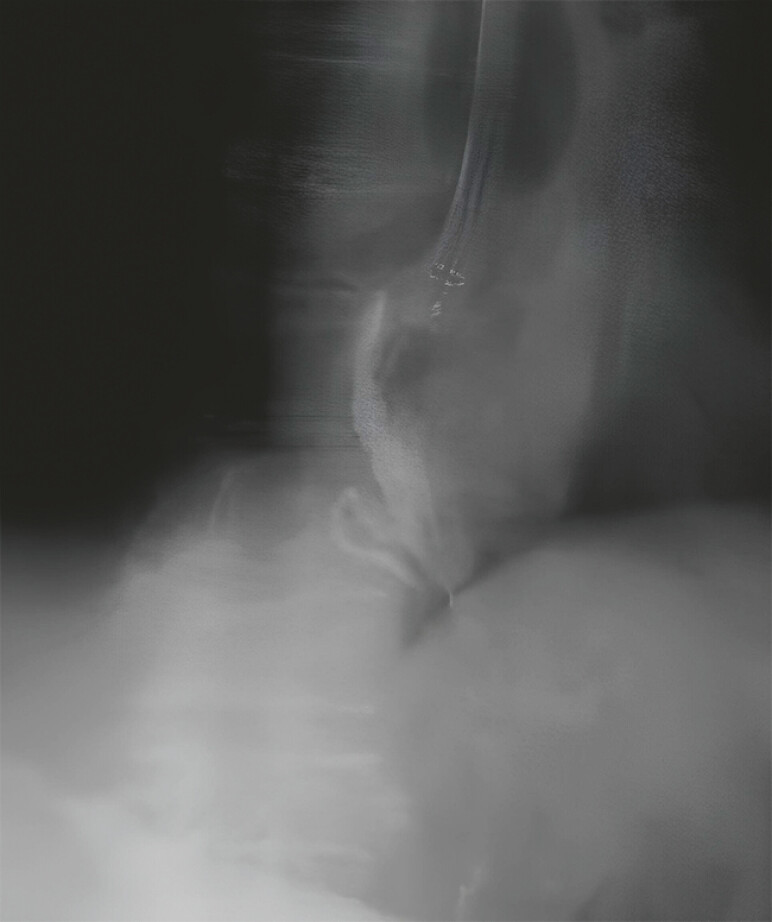
During fluoroscopy, the scope tip is translucent, which allows clear identification of the balloon and ethanolamine oleate flow.

Recently, EVL has been reported as commonly leading to recurrence when used alone 3 . By contrast, EISL, a combination of EIS and EVL, is reportedly useful as a treatment combining embolization of the blood supply by EIS and local blood flow blockage by EVL 4 . The feature of the single-use scope demonstrably makes it possible to perform EVL/EIS both safely and effectively.

Endoscopy_UCTN_Code_TTT_1AO_2AD

## Notes

Endoscopy E-Videos https://eref.thieme.de/e-videos
*E-Videos* is an open access online section of the journal *Endoscopy* , reporting on interesting cases and new techniques in gastroenterological endoscopy. All papers include a high-quality video and are published with a Creative Commons CC-BY license. Endoscopy E-Videos qualify for HINARI discounts and waivers and eligibility is automatically checked during the submission process. We grant 100% waivers to articles whose corresponding authors are based in Group A countries and 50% waivers to those who are based in Group B countries as classified by Research4Life (see: https://www.research4life.org/access/eligibility/ ). This section has its own submission website at https://mc.manuscriptcentral.com/e-videos .
